# The N270 as an index of consumer commodity color preference in the S1–S2 paradigm

**DOI:** 10.3389/fpsyg.2024.1350358

**Published:** 2024-06-07

**Authors:** Deming Shu, Dianzhi Liu, Gong-Liang Zhang

**Affiliations:** ^1^School of Education, Soochow University, Suzhou, China; ^2^The Autism Research Center, Soochow University, Suzhou, China

**Keywords:** N270, commodity, color, preference, neuromarketing

## Abstract

**Introduction:**

Affective decision-making is a prominent topic in consumer psychology research, with its core assumption being that consumers tend to purchase brands and commodities they like. However, the reasons behind why we develop emotional responses of liking or disliking toward certain commodities, as well as what the underlying neural mechanisms are, remain largely unknown.

**Methods:**

This study utilized the S1–S2 paradigm in an experiment wherein S1 presented 12 types of commodities and S2 displayed 48 distinct colored squares. Participants were instructed to assess whether they “Like” or “Dislike” the commodity in S1, which was colored with the S2 color. Electroencephalogram (EEG) recordings were obtained during the reaction process and subsequently analyzed to examine the components of S2-induced event-related potentials (ERPs).

**Results:**

The analysis revealed that S2 elicited a significant N270 in the prefrontal scalp area under both the “Like” and “Dislike” conditions. Interestingly, the amplitude of the N270 was significantly higher during the “Dislike” condition compared to the “Like” condition.

**Discussion:**

The N270 component was shown to reflect the conflict in physical attributes between S1 and S2, as well as the conflict between commodity and color. This highlights the potential utility of this component as an objective EEG indicator of consumer commodity color preferences in future marketing research.

## 1 Introduction

Sales companies typically gather extensive insights into consumer preferences through methods such as video surveillance, satisfaction surveys, focus groups, and other similar approaches. Recent studies have demonstrated that analyzing consumer brainwave patterns can partially reveal consumer reactions to advertising, brand messages, and relevant commercial information (Izadi et al., 2021; Mashrur et al., 2022; Shah et al., 2022; Kislov et al., 2023; Ouzir et al., 2024). These studies provide empirical evidence of effective strategies and pitfalls in the retail domain, with some also presenting evidence from objective neuroelectrophysiological indicators alongside subjective evaluations. Color usage in marketing is a fundamental aspect of design that, significantly influences consumer behavior and purchase attitudes (Li et al., 2020; Martinez et al., 2021); even infants have been observed to demonstrate a color preference for color (Zemach et al., 2007; Skelton and Franklin, 2020; Mcadams et al., 2023). Despite the importance of color in marketing, previous studies have primarily used behavioral tasks to investigate consumers’ color preference in commodities. However, behavioral data may lack the sensitivity to capture the intricate nuances of color preference and are contingent upon certain levels of subjectivity. Consequently, the physiological components of color preference in commodities remain underexplored.

Consumer decision-making is a fundamental aspect of consumer behavior, and it can be categorized into three types: cognitive decision-making, habitual decision-making, and affective decision-making. Affective decision-making is a widely debated topic in consumer psychology research, referring to the decisions made by consumers when purchasing goods or services influenced by emotional factors. These decisions can be influenced by factors such as the consumer’s emotional state, emotional preferences, emotional experiences, and emotional evaluations. Likes/dislikes represent a form of affect, expressing an individual’s emotional response to an object or event, which may be based on personal preferences, sensory enjoyment, or emotional experiences. The role of affect in purchase decisions has been emphasized in previous research (Morrison and Crane, 2007), particularly when there is insufficient cognitive information to differentiate between alternative products, affect can offer additional information (Peterson et al., 1986).

A fundamental assumption in marketing research is that consumers tend to purchase brands and commodities they like. What factors contribute to the development of liking or disliking affective responses toward specific commodities? What is the relationship between affect and cognition? What are the neural mechanisms underlying affect generation? These questions still lack clear answers. Early hypotheses suggest that consumers’ affective and cognitive systems operate independently, with affect being directly triggered by the features of target stimuli, without necessarily requiring cognition to elicit affective responses (Oliver, 1993). This perspective primarily emphasizes the impact of esthetic experiences rather than focusing solely on the functional advantages offered by commodities. Additionally, consumers’ imagined product features can also elicit affective responses and influence purchasing decisions (Phillips et al., 1995), referred to as anticipated or expected affect (Loewenstein and Lerner, 2003). Similarly, in the field of consumer satisfaction research, this perspective is referred to as the expectancy-disconfirmation model (Rust and Oliver, 1993), which proposes that customer satisfaction is influenced by the disparity between customer expectations and the actual product or service experience. However, affect and emotion are intricately interconnected systems (Corrêa, 2024). Although involving distinct brain regions, they are closely connected through neural pathways. Therefore, the cognitive-affective model (Menon and Dubé, 2000), in contrast to the independence hypothesis, has gained increasing acceptance within the academic community. This model suggests that consumers develop affective states based on cognitive processing of the commodity, ultimately influencing their decision-making. In other words, affect serves as a mediating variable between cognitive processing and consumption decisions.

This study investigates how cognition triggers emotional responses by using the color attribute of a commodity as a feature. When evaluating an object, people’s initial reactions are often based on the object’s color. Color is therefore one of the most elementary techniques in marketing, and its relevant application produces behavioral effects that reflect purchase attitudes (Li et al., 2020; Martinez et al., 2021). To explore the underlying mechanism behind affective responses, we propose an intuitive hypothesis: consumers evaluate whether they like a commodity by comparing it with the mental representation of the commodity in their minds. To test this hypothesis, we adopted an S1-S2 experimental paradigm. Participants were first presented with a commodity name (S1), followed by the presentation of a color (S2), and they had to judge whether they liked the color of the commodity. Furthermore, we hypothesize that the commodity name in S1 would lead participants to form a mental representation of the commodity based on their existing memories. In the S2 presentation, participants would compare the presented color with the color in their mental representation and make an evaluation of “Like” if the two colors were consistent, or “Dislike” if they were not. We chose an event-related potential (ERP) component called N270 as an objective measure to validate this hypothesis. The utility of neuroscience tools in capturing neural signals corresponding to mental activity has been demonstrated; these techniques facilitate the comprehension of the underlying neural mechanisms behind customers’ behavior (Ma et al., 2021).

The N270 is a negative ERP component with a peak latency of around 270 ms, elicited when there is a mismatch between the physical and social attributes of the first and second stimuli (Cao et al., 2020; Galigani et al., 2021; Wang et al., 2021; Zhou et al., 2021; Gómez-Velázquez et al., 2022). The N270 was elicited through a range of stimuli, including shape, color, spatial location, and simple arithmetic tasks (Bennett et al., 2014). This N270 component is the electric signal of the cerebral cortex for processing conflict information, and the component exhibits characteristics analogous to those of other negative ERP components, such as error-related negativity (Figuracion et al., 2023; Meyer et al., 2023), mismatch negativity (Riel et al., 2022; Usui et al., 2023), and semantic N400 (Undorf et al., 2020; Hsin et al., 2023). Therefore, if there is a significant difference in the N270 component between the “Like” trials and the “Dislike” trials, the participants’ assessment of “Like”/ “Dislike” is associated with the consistency between the color of stimulus S2, and the color representation induced by the memory of stimulus S1.

## 2 Materials and methods

### 2.1 Participants

This study included a sample of 15 Chinese college students (seven men, eight women), all right-handed and with an age range of 20–26 years (mean = 21.7 ± 1.77 years). The participants had either accurate or corrected-to-normal visual acuity, none exhibited color blindness or weakness, and none had a recorded history of neurological or mental disorders. The local Ethics Committee approved the research protocol.

### 2.2 Materials

The prime stimuli (S1) comprised 12 types of commodities that were presented in white Chinese characters and a black background. Six types of commodities were selected from each of the following two categories: (i) household appliances, namely washing machines, refrigerators, air conditioners, electric fans, rice cookers, and watering troughs; and (ii) textiles, namely sofas, quilt covers, curtains, towels, trousers, and shirts. The experiment was conducted in Chinese, and the names of these commodities in Chinese include only two or three characters.

The target stimuli (S2) comprised 48 colors selected based on Hue (0, 60, 120, 180), Saturation (60, 120, 180), and Lightness (48, 96, 144, 192). The colored squares measuring 10 × 10 cm were presented against a black background. Accordingly, 12 different types of commodities were paired with 48 distinct colored squares to create 576 pairs of stimuli, with each pair consisting of a commodity name (S1) and a colored square (S2).

**Figure 1:**
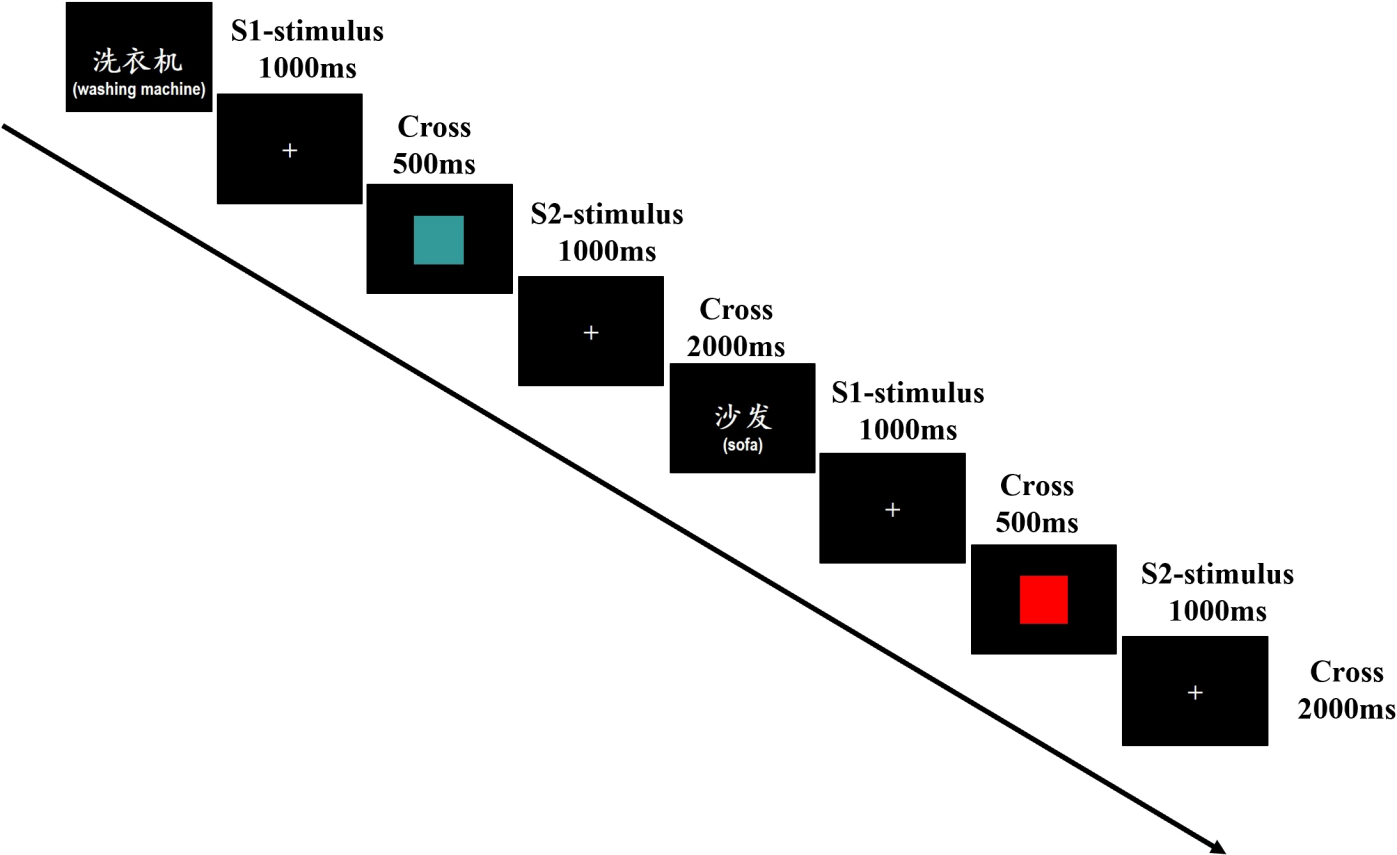
Schematic representation of the experimental procedure. The contents within the parentheses are not displayed during the formal experiment.

The visual stimuli were presented to each participant at the center of the screen of a computer-controlled video monitor. The stimuli, S1 or S2, were consistently presented at the fixation point for 1,000 ms each. S1 was displayed at a visual angle of either 1.48° × 3° (two Chinese characters) or 1.48° × 4.63° (three Chinese characters), and S2 at a visual angle of 4.77° × 4.77°. The interstimulus interval was set at 500 ms, and there was a 2,000 ms interval between the termination of a S2 and the onset of the subsequent S1. The stimuli pairs (S1–S2) were presented in a randomized sequence with equal probability. The experimental procedure is schematically depicted in Figure 1.

### 2.3 Procedure

The electroencephalogram (EEG) was continuously recorded using a Neuroscan Synamp2 Amplifier (Neurosoft Labs, Inc.) with a bandpass filter set at 0.05–100 Hz and a sampling rate of 1,000 Hz. An electrode cap equipped with 64 Ag/AgCl electrodes was arranged following the extended international 10–20 system and referenced to linked mastoids. The vertical and horizontal electrooculograms were recorded using two pairs of electrodes: one positioned above and below the right eye, and the other placed 10 mm away from the lateral canthi. The electrode impedance was consistently maintained below 5kΩ throughout the whole experiment.

After electrode application, the participants were instructed to sit on a comfortable sofa situated in a shielded room and focus their gaze on a fixed point at the center of a computer display positioned 1.2 m away from their eyes. Participants were instructed to indicate their preference for purchasing the commodity presented in the first stimulus, which was colored with the subsequent color. They were required to promptly evaluate the stimuli by pressing one of two buttons in a push pad; they were instructed to press the left button if they believed the commodity was suitable for purchase (i.e., a favorable color–commodity match; a “Like” response), and the right button otherwise (i.e., a “Dislike” response). The participants were divided into two groups, with one group using their left hand and the other group using their right hand for the “Like” response. After providing participants with 30 practice trials, 576 trials were conducted.

### 2.4 EEG analysis

EEG recordings were segmented from 200 ms before the onset of S2 (color squares) on the display to 1,000 ms after the onset, with the first 200 ms pre-stimulus being the baseline. Trials contaminated by amplifier clipping, bursts of electromyographic activity, or peak-to-peak deflection exceeding ±100μv were excluded from averaging. The remaining trials were baseline-corrected. The EEG segments corresponding to different color squares were averaged based on participants’ responses (i.e., “Like” and “Dislike” ratings). The averaged ERPs were digitally filtered using a low-pass filter at 30 Hz (24 dB/octave), as this study focused primarily on the analysis of ERPs elicited by conditions associated with “Like” and “Dislike.”

Six electrode sites (F5, FZ, F4, FC3, FCZ, and FC4) were selected for further analysis. The selection of these electrode sites was based on a comprehensive analysis that considered previous findings indicating the prefrontal localization of N270 (Fan and Zhang, 2019; Zhao et al., 2020; Rivera and Soylu, 2021). Further, the selection was supported by examining grand-averaged ERPs for both the “Like” and “Dislike” conditions as well as EEG topographic maps. A statistical analysis was performed involving the P200 (160–220 ms), N270 (240–330 ms), and N400 (380–450 ms) ERP components at the frontal and frontal-central sites. The latencies and amplitudes of the P200, N270, and N400 components were analyzed using two-way repeated measures analysis of variance (ANOVA) considering the two conditions (“Like” and “Dislike”) and six electrode sites (F5, FZ, F4, FC3, FCZ, and FC4). The degrees of freedom for the F-ratio were adjusted using the Greenhouse–Geisser method.

## 3 Results

### 3.1 Behavioral data

The behavioral data of 15 subjects were analyzed, revealing an average “Like” ratio of 0.48 ± 0.17 and a reaction time of 771.06 ± 251.75 ms. Similarly, the average “Dislike” ratio was 0.52 ± 0.17, with an average reaction time of 807.28 ± 287.28 ms. Paired-sample *T*-tests indicated that there were no significant differences in both the average ratio [*t*(14) = −0.43, *p* = 0.673, *Cohen’s d* = 0.12] and response time [*t*(14) = −1.27, *p* = 0.225, *Cohen’s d* = 0.33] between “Like” and “Dislike.”

### 3.2 ERP results

**Figure 2:**
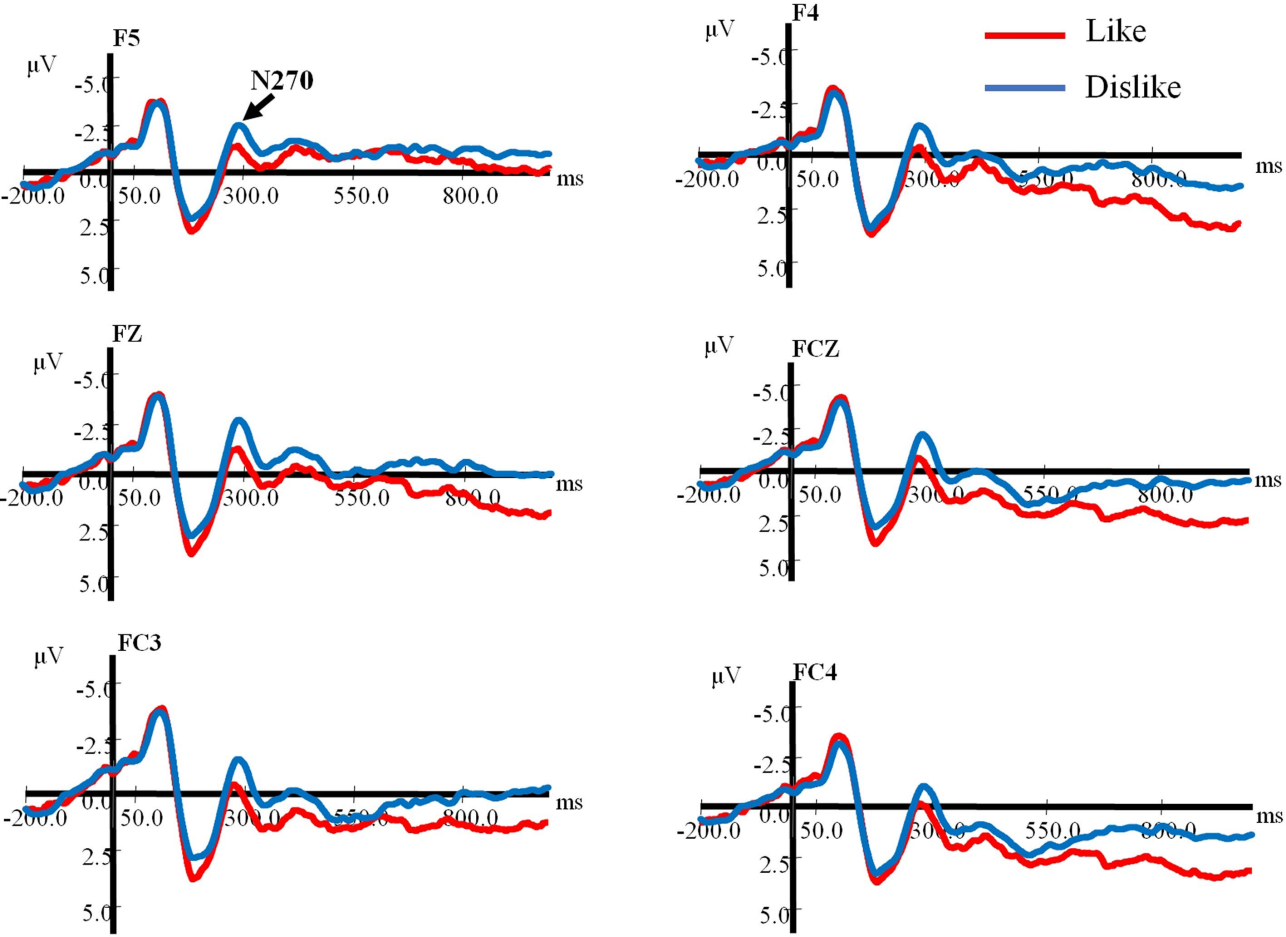
Grand-averaged event-related potentials for the “Like” and “Dislike” conditions regarding the electrodes in frontal areas.

**Figure 3:**
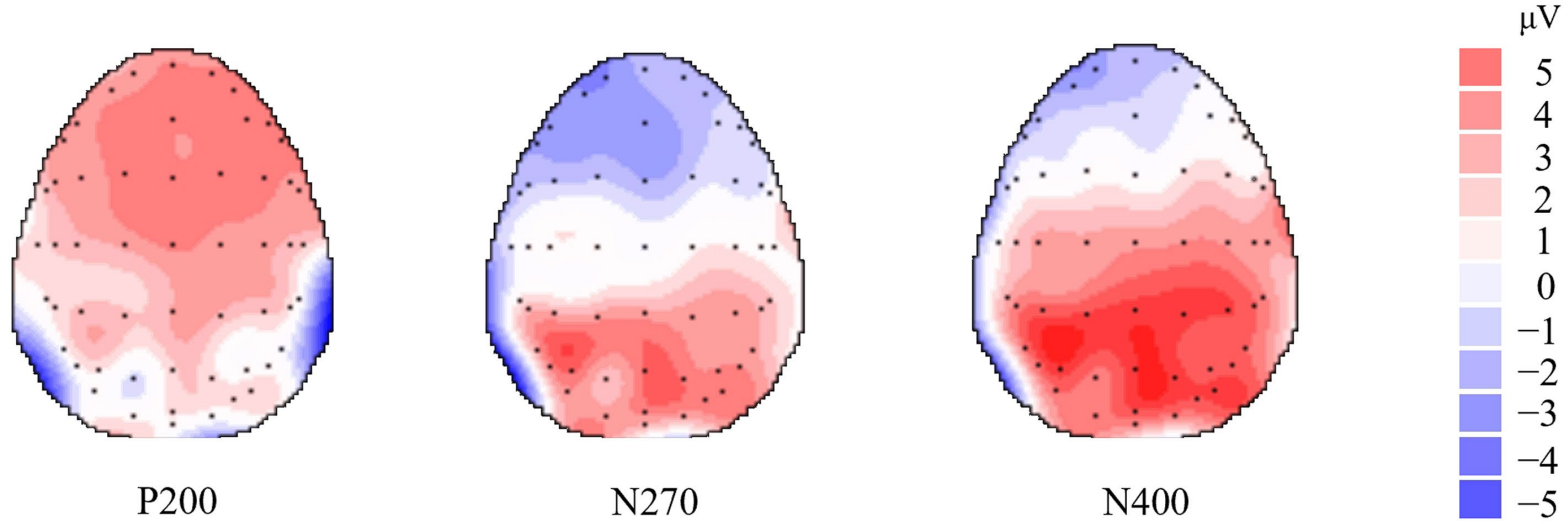
Topographical distributions of P200 (184 ms), N270 (288 ms) and N400 (427 ms).

Following the probe colored-square onset, a remarkable positive wave, P200, was recorded from widespread scalp areas in two different conditions. N270 was most prominent at the frontal sites and analyzed at F5, FZ, F4, FC3, FCZ, and FC4. In addition, the N400 component was prominently observed at the frontal sites in both conditions. The N270 component was identified in the interval between 240 and 330 ms, the N400 component was identified in the interval between 380 and 450 ms, and the P200 component was identified in the interval between 160 and 220 ms. The grand-averaged ERPs for the “Like” and “Dislike” conditions in relation to frontal electrode sites are depicted in Figure 2. Figure 3 illustrates the topographical distributions of P200 (184 ms), N270 (288 ms), and N400 (427 ms). To examine the effect of the commodity–color conflict processes on the principal negative component, a repeated-measures ANOVA of peak amplitudes and latencies was conducted for N270; specifically, 2 (Condition: “Like,” “Dislike”) * 6 (Electrode: F5, FZ, F4, FC3, FCZ, and FC4). The ANOVA for the peak amplitudes of N270 showed a main effect, *F* (1, 14) = 144.85, *p* < 0.001, $\eta_{p}^{2}$ = 0.910. The simple effects analysis showed that the N270 was more negative for “Dislike” (−2.52 ± 2.99 μV) than for “Like” (−1.33 ± 3.24 μV), *p* < 0.001. There was also a main effect of the latencies of N270 [*F* (1, 14) = 5.68, *p* = 0.032 $\eta_{p}^{2}$ = 0.290]. The simple effects analysis showed slower N270 latencies for “Dislike” (289.73 ± 15.77 ms) than for “Like” (283.51 ± 17.20 ms). Both the main effect of the Electrode and the interaction between the Condition and Electrode were not significant.

We further conducted a repeated-measures ANOVA of peak amplitudes and latencies for the P200; however, there was no main effect, with the mean amplitude of “Dislike” and “Like” being 3.89 ± 3.12 μV and 4.53 ± 3.18 μV, respectively, *F* (1, 14) = 4.20, *p* = 0.060, $\eta_{p}^{2}$ = 0.231; the mean latency of “Dislike” and “Like” was 193.12 ± 21.39 ms and 192.22 ± 16.58 ms, respectively, *F* (1, 14) = 0.10, *p* = 0.755, $\eta_{p}^{2}$ = 0.007.

## 4 Discussion

The present study aims to investigate the neural mechanisms underlying consumers’ affective decision-making using electroencephalography (EEG) technology. It was hypothesized that consumers assess their preference for a product by comparing it to their internal impression of the product. To validate this hypothesis, we adopted an S1-S2 paradigm and utilized the ERP N270 component as an objective neurophysiological indicator. The findings of this study confirm our hypothesis, as the amplitude of N270 was significantly higher in the “Dislike” condition compared to the “Like” condition. The N270 component is typically evoked when there is a mismatch between the physical and social attributes of the initial and subsequent stimuli (Cao et al., 2020; Galigani et al., 2021; Wang et al., 2021; Zhou et al., 2021; Gómez-Velázquez et al., 2022). Thus, this result suggests a greater discrepancy between the S2 color and the subjects’ internal representation of the color of the S1 commodity when the evaluation of the commodity was “Dislike.” This suggests that, within the S1-S2 paradigm, N270 may serve as an objective neurophysiological indicator for consumers’ affective decision-making.

In this study, participants were asked to evaluate whether they liked the S1 commodity in the S2 color. Before making their evaluations, participants may have also undergone expectancy-disconfirmation cognitive processing. When the S1 commodity appeared, participants’ mental representations served as their expectations. If the S2 color was inconsistent with this expectation, expectancy-disconfirmation occurred, leading participants to make a “Dislike” evaluation. Therefore, the results of this study indicate that cognitive processing of commodities influences affective evaluations, which aligns with the cognitive-affective model and suggests that emotional responses may serve as mediating variables between cognitive processing and consumer decisions (Menon and Dubé, 2000). It should be noted that the expectancy-disconfirmation in the S1-S2 paradigm is slightly different from the expectancy-disconfirmation in the expectancy disconfirmation model. In the expectancy disconfirmation model, expectancy-disconfirmation refers to the difference between the actual performance experience of a product and the expectation, whereas in the S1-S2 paradigm, it pertains to the difference between the product’s features (e.g., color) and consumers’ anticipation of that feature. Worth noting is that this study uncovers the neural mechanism of expectancy-disconfirmation and proposes that the ERP N270 component can serve as a neurophysiological indicator of expectancy-disconfirmation.

In this study, we found that the N270 component was significantly larger in the “Dislike” condition compared to the “Like” condition. This result indicates a greater mismatch in the “Dislike” condition as the N270 component is typically elicited in response to a mismatch between the physical and social attributes of the first and second stimuli. Specifically, when participants evaluated the commodity as “Dislike,” there was a greater mismatch between their psychological representation of the color of the first stimulus (S1) and the color of the second stimulus (S2). One possible explanation for the relationship between mismatch and preference evaluation is the role of familiarity as a mediator. Our brains tend to prefer familiar things because familiarity implies that they have left some psychological representation in the brain, making processing easier (Rhodes, 2006). For example, research has shown that familiarity influences adults’ and infants’ facial feature preferences (Park et al., 2010), and plays a crucial role in forming judgments, preferences, and social interactive attitudes toward others (Antonius et al., 2013). Moreover, familiarity with food can influence people’s food preferences evaluation (Torrico et al., 2019). Aligned with these studies, familiarity can explain the preference evaluation of the participants in this study: when colors matched the participant’s mental representation of the product, it indicated familiarity with the product in that particular color, leading to a “Like” evaluation; conversely, if there was a mismatch, participants evaluated it as “Dislike.”

N400 was originally discovered in a semantic experiment in which sentences had incongruent endings (Ralph et al., 2020; Delogu et al., 2021; Pélissier and Ferragne, 2022; Toffolo et al., 2022); in these semantic experiments, when a participant responded to the incongruous semantic context of the sentence stimuli, N400 appeared. We also observed the N400 component following the late positive component, which appeared subsequently to the N270 component. Therefore, it is plausible that the N400 component in this study was elicited by conflicts arising from the multiple dimensions of colors and commodities.

Our findings also showcase a non-significant difference for the P200 component between the “Like” and “Dislike” conditions. The non-significant amplitude of P200 under the two conditions and the lack of a substantial effect size have precluded an in-depth discussion thereof. We are uncertain whether P200 can be considered a valuable ERP component associated with consumer commodity color preferences. This showcases a future pathway for experimental investigations in this field.

Despite its clear contributions, this study has several limitations that must be addressed in future research. The first limitation of our research was the small sample size. Due to the small sample size, we observed only trends of differences in the P200 component between the “Like” and “Dislike” conditions, but they were not statistically significant. Fortunately, P200 was not the ERP component of primary interest in this study. Second, as our study used a quasi-experimental design, the “Like” and “Dislike” conditions were not randomly assigned, limiting our ability to draw a conclusion on causal relationships. Future research could consider using a true experimental design to explore the causal relationship between these conditions. Finally, as this study did not directly measure the variable of familiarity, we were unable to verify whether familiarity mediated the relationship between mismatch and preference evaluation. Future research could incorporate measures of familiarity to test this hypothesis.

## 5 Conclusion

This study demonstrates that the N270 ERP component can be elicited by physical conflicts between S1 and S2 and consumer commodity color preferences. Interestingly, greater N270 amplitudes were observed when disliked colors were presented. The findings demonstrate that companies can employ the N270 component in marketing research and commodity design as an index of consumers’ color preferences for commodities.

## Data availability statement

The original contributions presented in the study are included in the article/Supplementary material, further inquiries can be directed to the corresponding authors.

## Ethics statement

The studies involving humans were approved by the Ethics Committees of School of Education, Soochow University. The studies were conducted in accordance with the local legislation and institutional requirements. The participants provided their written informed consent to participate in this study.

## Author contributions

DS: Methodology, Writing – original draft, Writing – review & editing. DL: Project administration, Writing – review & editing. G-LZ: Project administration, Writing – review & editing.

## References

[ref1] Antonius D. Bruce K. L. Moisa B. Sinclair S. J. Malaspina D. Tremeau F. (2013). Familiarity preference in schizophrenia is associated with ambivalent attitudes towards others. Schizophr. Res. 150, 229–234. doi: 10.1016/j.schres.2013.07.056, PMID: 23954145

[ref2] Bennett M. A. Duke P. A. Fuggetta G. (2014). Event-related potential N270 delayed and enhanced by the conjunction of relevant and irrelevant perceptual mismatch. Psychophysiology 51, 456–463. doi: 10.1111/psyp.12192, PMID: 24611511

[ref3] Cao S. Wang Y. Z. Wang H. L. Chen H. J. Zhang G. H. Kritikos A. (2020). A facilitatory effect of perceptual incongruity on target-source matching in pictorial metaphors of Chinese advertising: EEG evidence. Adv. Cogn. Psychol. 16, 1–12. doi: 10.5709/acp-0279-z, PMID: 32537039 PMC7278523

[ref4] Corrêa C. G. L. (2024). The relation between affection and cognition: theoretical perspectives. Psicologia Escolar e Educacional 28, 1–9. doi: 10.1590/2175-35392024-257346-t, PMID: 37925091

[ref5] Delogu F. Brouwer H. Crocker M. W. (2021). When components collide: spatiotemporal overlap of the N400 and P600 in language comprehension. Brain Res. 1766:147514. doi: 10.1016/j.brainres.2021.147514, PMID: 33974906

[ref6] Fan B. Zhang Q. R. (2019). Does the aura surrounding healthy-related imported products fade in China? ERP evidence for the country-of-origin stereotype. PLoS One 14:e0216866. doi: 10.1371/journal.pone.0216866, PMID: 31120899 PMC6532883

[ref7] Figuracion M. T. Kozlowski M. B. Macknyk K. S. Heise M. B. Pieper S. M. Alperin B. R.. (2023). The relationship between emotion dysregulation and error monitoring in adolescents with ADHD. Res. Child Adolesc. Psychopathol. 52, 605–620. doi: 10.1007/s10802-023-01127-z 37843650 PMC11660821

[ref8] Galigani M. Ronga I. Bruno V. Castellani N. Sebastiano A. R. Fossataro C.. (2021). Face-like configurations modulate electrophysiological mismatch responses. Eur. J. Neurosci. 53, 1869–1884. doi: 10.1111/ejn.15088, PMID: 33332658

[ref9] Gómez-Velázquez F. R. González-Garrido A. A. Ruiz-Stovel V. D. Villuendas-González E. R. Martínez-Ramos A. Altamirano-Ríos M. (2022). Event-related brain potentials study of arithmetic fact retrieval in children with different math achievement levels. J. Cogn. Psychol. 34, 996–1010. doi: 10.1080/20445911.2022.2090571

[ref10] Hsin C. Chao P. Lee C. (2023). Speech comprehension in noisy environments: evidence from the predictability effects on the N400 and LPC. Front. Psychol. 14:1105346. doi: 10.3389/fpsyg.2023.1105346, PMID: 36874840 PMC9974639

[ref11] Izadi B. Ghaedi A. Ghasemian M. (2021). Neuropsychological responses of consumers to promotion strategies and the decision to buy sports products. Asia Pac. J. Market. Logist. 34, 1203–1221. doi: 10.1108/APJML-01-2021-0026

[ref12] Kislov A. Gorin A. Konstantinovsky N. Klyuchnikov V. Bazanov B. Klucharev V. (2023). Central EEG beta/alpha ratio predicts the population-wide efficiency of advertisements. Brain Sci. 13:57. doi: 10.3390/brainsci13010057 PMC9856603 36672039

[ref13] Li S. T. Chen R. R. Yang L. J. Huang D. L. Huang S. M. (2020). Predictive modeling of consumer color preference: using retail data and merchandise images. J. Forecast. 39, 1305–1323. doi: 10.1002/for.2689

[ref14] Loewenstein G. Lerner J. S. (2003). “The role of affect in decision making” in Handbook of affective science, vol. 619, 3.

[ref15] Ma Q. G. Wang M. L. Da Q. (2021). The effects of brand familiarity and product category in brand extension: an ERP study. Neurosci. Res. 169, 48–56. doi: 10.1016/j.neures.2020.06.010, PMID: 32652108

[ref16] Martinez L. M. Rando B. Agante L. Abreu A. M. (2021). True colors: Consumers' packaging choices depend on the color of retail environment. J. Retail. Consum. Serv. 59:102372. doi: 10.1016/j.jretconser.2020.102372

[ref17] Mashrur F. R. Rahman K. M. Miya M. T. I. Vaidyanathan R. Anwar S. F. Sarker F.. (2022). An intelligent neuromarketing system for predicting consumers' future choice from electroencephalography signals. Physiol. Behav. 253:113847. doi: 10.1016/j.physbeh.2022.113847, PMID: 35594931

[ref18] Mcadams P. Chambers M. Bosten J. M. Skelton A. E. Franklin A. (2023). Chromatic and spatial image statistics predict infants' visual preferences and adults' aesthetic preferences for art. J. Vis. 23:2. doi: 10.1167/jov.23.8.2, PMID: 37526623 PMC10399602

[ref19] Menon K. Dubé L. (2000). Ensuring greater satisfaction by engineering salesperson response to customer emotions. J. Retail. 76, 285–307. doi: 10.1016/S0022-4359(00)00034-8

[ref20] Meyer A. Chong L. Wissemann K. Mehra L. Mirzadegan I. (2023). An experimental therapeutics approach to the development of a novel computerized treatment targeting error-related brain activity in young children. Behav. Ther. 54, 652–665. doi: 10.1016/j.beth.2023.01.005, PMID: 37330255

[ref21] Morrison S. Crane F. G. (2007). Building the service brand by creating and managing an emotional brand experience. J. Brand Manag. 14, 410–421. doi: 10.1057/palgrave.bm.2550080

[ref22] Oliver R. L. (1993). Cognitive, affective, and attribute bases of the satisfaction response. J. Consum. Res. 20, 418–430. doi: 10.1086/209358

[ref23] Ouzir M. Chakir Lamrani H. Bradley R. L. El Moudden I. (2024). Neuromarketing and decision-making: classification of consumer preferences based on changes analysis in the EEG signal of brain regions. Biomed. Signal Process. Control 87:105469. doi: 10.1016/j.bspc.2023.105469

[ref24] Park J. Shimojo E. Shimojo S. (2010). Roles of familiarity and novelty in visual preference judgments are segregated across object categories. Proc. Natl. Acad. Sci. USA 107, 14552–14555. doi: 10.1073/pnas.1004374107, PMID: 20679235 PMC2930416

[ref25] Pélissier M. Ferragne E. (2022). The N400 reveals implicit accent-induced prejudice. Speech Comm. 137, 114–126. doi: 10.1016/j.specom.2021.10.004

[ref26] Peterson R. A. Hoyer W. D. Wilson W. R. (1986). The role of affect in consumer behavior: Emerging theories and applications: Papers from a symposium held September 21–22, 1984 at the University of Texas at Austin and sponsored by the college of business administration and the IC2 Institute of the University of Texas at. Austin: Lexington Books.

[ref27] Phillips D. M. Olson J. C. Baumgartner H. (1995). Consumption visions in consumer decision making. ACR North Am. Adv. 22, 280–284.

[ref28] Ralph Y. K. Schneider J. M. Abel A. D. Maguire M. J. (2020). Using the N400 event-related potential to study word learning from context in children from low-and higher-socioeconomic status homes. J. Exp. Child Psychol. 191:104758. doi: 10.1016/j.jecp.2019.104758, PMID: 31855830 PMC8191850

[ref29] Rhodes G. (2006). The evolutionary psychology of facial beauty. Annu. Rev. Psychol. 57, 199–226. doi: 10.1146/annurev.psych.57.102904.190208, PMID: 16318594

[ref30] Riel H. MacPhee C. Rudolph E. D. Tibbo P. G. Fisher D. J. (2022). MMN and P3a elicited by a novelty paradigm in healthy controls: an investigation of sex differences. Neurosci. Lett. 781:136654. doi: 10.1016/j.neulet.2022.136654, PMID: 35469822

[ref31] Rivera B. Soylu F. (2021). Incongruity in fraction verification elicits N270 and P300 ERP effects. Neuropsychologia 161:108015. doi: 10.1016/j.neuropsychologia.2021.108015, PMID: 34474064

[ref32] Rust R. T. Oliver R. L. (1993). Service quality: New directions in theory and practice. Los Angeles, CA: SAGE Publications.

[ref33] Shah S. M. A. Usman S. M. Khalid S. Rehman I. U. Anwar A. Hussain S.. (2022). An ensemble model for consumer emotion prediction using EEG signals for neuromarketing applications. Sensors 22:9744. doi: 10.3390/s22249744, PMID: 36560113 PMC9782208

[ref34] Skelton A. E. Franklin A. (2020). Infants look longer at colours that adults like when colours are highly saturated. Psychon. Bull. Rev. 27, 78–85. doi: 10.3758/s13423-019-01688-5, PMID: 31848908 PMC7000485

[ref35] Toffolo K. K. Freedman E. G. Foxe J. J. (2022). Evoking the N400 event-related potential (ERP) component using a publicly available novel set of sentences with semantically incongruent or congruent eggplants (endings). Neuroscience 501, 143–158. doi: 10.1016/j.neuroscience.2022.07.030, PMID: 35964833 PMC9540983

[ref36] Torrico D. D. Fuentes S. Gonzalez V. C. Ashman H. Dunshea F. R. (2019). Cross-cultural effects of food product familiarity on sensory acceptability and non-invasive physiological responses of consumers. Food Res. Int. 115, 439–450. doi: 10.1016/j.foodres.2018.10.054, PMID: 30599962

[ref37] Undorf M. Amaefule C. O. Kamp S. (2020). The neurocognitive basis of metamemory: using the N400 to study the contribution of fluency to judgments of learning. Neurobiol. Learn. Mem. 169:107176. doi: 10.1016/j.nlm.2020.107176, PMID: 32001337

[ref38] Usui K. Kirihara K. Araki T. Tada M. Koshiyama D. Fujioka M.. (2023). Longitudinal change in mismatch negativity (MMN) but not in gamma-band auditory steady-state response (ASSR) is associated with psychological difficulties in adolescence. Cereb. Cortex 33, 11070–11079. doi: 10.1093/cercor/bhad346, PMID: 37815245 PMC10631957

[ref39] Wang Y. H. Bao W. Luo J. L. (2021). When old information is intermixed with new elements: an event-related potential study. Biol. Psychol. 163:108132. doi: 10.1016/j.biopsycho.2021.108132, PMID: 34098043

[ref40] Zemach I. Chang S. Teller D. Y. (2007). Infant color vision: prediction of infants' spontaneous color preferences. Vision Res. (Oxford) 47, 1368–1381. doi: 10.1016/j.visres.2006.09.024, PMID: 17118421

[ref41] Zhao F. Yang C. Zheng Y. (2020). The N270 in facial S1-S2 paradigm as a biomarker for children with attention-deficit/hyperactivity disorder. IEEE Access 8, 88969–88976. doi: 10.1109/ACCESS.2020.2993058

[ref42] Zhou Q. H. Song P. H. Wang X. M. Lin H. Wang Y. P. (2021). Transcranial magnetic stimulation over the right posterior superior temporal sulcus promotes the feature discrimination processing. Front. Hum. Neurosci. 15:663789. doi: 10.3389/fnhum.2021.663789, PMID: 34220471 PMC8253362

